# Rare Anemias: Are Their Names Just Smoke and Mirrors?

**DOI:** 10.3389/fphys.2021.690604

**Published:** 2021-06-10

**Authors:** Greta Simionato, Richard van Wijk, Stephan Quint, Christian Wagner, Paola Bianchi, Lars Kaestner

**Affiliations:** ^1^Institute for Clinical and Experimental Surgery, Campus University Hospital, Saarland University, Homburg, Germany; ^2^Experimental Physics, Dynamics of Fluids Group, Saarland University, Saarbrücken, Germany; ^3^Central Diagnostic Laboratory - Research, University Medical Center Utrecht, Utrecht University, Utrecht, Netherlands; ^4^Cysmic GmbH, Saarbrücken, Germany; ^5^Physics and Materials Science Research Unit, University of Luxembourg, Luxembourg, Luxembourg; ^6^Fondazione Instituto di Ricovero e Cura a Carattere Scientifico Ca' Granda Ospedale Maggiore Policlinico Milano, Unità Operativa Complessa Ematologia, Unità Operativa Semplice Fisiopatologia delle Anemie, Milan, Italy; ^7^Theoretical Medicine and Biosciences, Campus University Hospital, Saarland University, Homburg, Germany

**Keywords:** rare anemias, red cell morphology, hereditary spherocytosis, dehydrated stomatocytosis, hereditary xerocytosis, Gárdos channelopathy

## Introduction

Numerous anemias and even neurodegenerative diseases are named after the predominant red blood cell (RBC) shape observed by microscopy - within this paper we refer to this as RBC morphology. Examples are spherocytes in hereditary spherocytosis (Huisjes et al., 2019), stomatocytes in hereditary stomatocytosis (Andolfo et al., 2018), elliptocytes in hereditary elliptocytosis (Soderquist and Bagg, 2013), sickle shaped deoxygenated RBCs in sickle cell disease (Cisneros and Thein, 2020), or acanthocytes in neuroacanthocytosis syndromes (Peikert et al., 2017).

A good portion of these names are justified because a substantial portion of the patient RBCs show at least under particular conditions the corresponding morphology. At the same time we have to admit that sometimes the shape classification can be confusing when, for example, sphero-ovalocytes (Jarolim et al., 1995), ovalocytes (Mohandas et al., 1984), elliptocytes (Motulsky et al., 1954), or poikilocytes (Agre et al., 1981) can all be attributed to the same disease: elliptocytosis.

The clinical evaluation of the cell shapes depends widely on the use of peripheral blood smears, i.e., a drop of fresh blood is smeared on a glass slide, dried, fixed and stained, a procedure that partly deteriorates the original cell morphology (Wenk, 1976). Extensive work was performed to characterize RBC shapes at electron microscopic super-resolution in 3D. These partly artistic images culminated in the seminal work by Marcel Bessis in the last century (Bessis, 1973). In the meantime, optical imaging technology progressed, e.g., confocal microscopy became widely available (Pawley, 2006) and was explored for the 3D-visualization of RBCs both in stasis (Khairy et al., 2008) and in flow (Quint et al., 2017). Optical technologies have less stringent requirements for sample preparation compared to electron microscopy (Abay et al., 2019). Confocal microscopy combined with automated processes including machine learning-based algorithms seems to lead to a revival of RBC visualization in 3D and the subsequent shape evaluation (Kaestner and Bianchi, 2020), shedding new lights on RBC morphological complexity. It is worthwhile to mention that 3D renderings based on confocal microscopy allow, in contrast to electron microscopy and probe scanning techniques, an unlimited 360° view.

Furthermore, RBC in their physiological environment are in constant flow and at least in capillary flow take completely different cell shapes as in stasis, e.g., discocytes are transformed into “croissants” or “slippers” in a flow speed dependent manner (Kihm et al., 2018). For most pathophysiologial conditions RBC morphology in (capillary) flow are not investigated, which is a so far missed diagnostic potential but at the same time is not in favor of naming anemias after RBC shapes in stasis.

Here we like to discuss examples of rare anemias named after the RBC shapes found in blood smears that are not representative of the disease condition based on both new microscopy applications and functional tests.

## Spherocyte Numbers in Hereditary Spherocytosis - Comparison Between 2D and 3D Images

**Figure 1:**
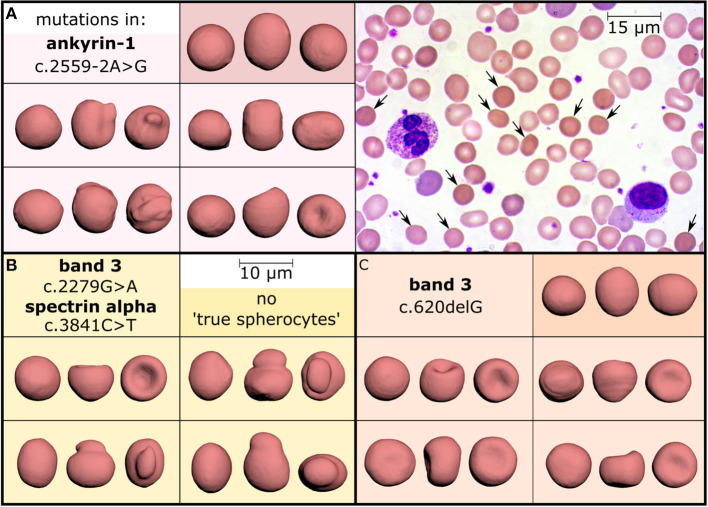
Investigation of hereditary spherocytosis red blood cell shapes. Three patients diagnosed with hereditary spherocytosis caused by different mutations (panels **A–C**) showed a spherocyte count of 11% **(A)**, 8% **(B)**, and 10% **(C)** in their stained peripheral blood smears, as exemplified in panel A (arrows, objective-magnification 100x). Comparison with 3D-rendered confocal recordings (objective-magnification 60x) of glutaraldehyde fixed and CellMask stained cells, however, demonstrated a different percentage of “true spherocytes”: 2.5% **(A)**, 0% **(B)**, and 0.08% **(C)**. They are visualized in the dark colored boxes, each showing one cell from three perpendicular directions and mostly reflecting the amount observed in healthy subjects (0–0.3%, examined in 15 donors). In contrast, many cells look like spherocytes from one direction (leftmost view in all boxes) but the other faces reveal different morphologies, such as mushroom-shaped cells, stomatocytes or other irregular-shaped cells (all light colored boxes) representing “pseudo spherocytes.” These observations could be confirmed in 10 hereditary spherocytosis patients after 3D-imaging of about 1,000 cells per subject. This Figure is a reprint of Simionato et al. (2021).

A recent study investigated the use of an artificial neural network to automatically recognize RBC shapes, proposing that the detailed 3D analysis may even identify the specific genetic defect causing a particular rare anemia (Simionato et al., 2021). As a side information, it was found that the spherocytes identified in peripheral blood smears from patients with hereditary spherocytosis are mostly just “pseudo spherocytes” (see Figure 1). The figure illustrates that information indeed gets lost in 2D images. Furthermore, the almost exclusive occurrence of “pseudo spherocytes” *per se* (not their number) seems to be independent of the particular mutation causing the hereditary spherocytosis.

This finding is actually not new, since Bessis already stated: “The cells it describes (spherocytes) are actually not spheres. They include a variety of cells which are etiologically and morphologically dissimilar. They have only one thing in common: an increase in their thickness” (Bessis, 1974). The scanning electron microscopy (SEM) allows a 3D surface scan (although not a 360° view), which allowed Bessis to make this statement and which is 100% confirmed by the 3D renderings based on confocal measurements (Figure 1).

## Dehydrated Stomatocytosis is a Terminology Contradiction in Itself

When referring to RBC dehydration, currently we face two completely different approaches. The traditional one refers to the RBC shapes. If cells dehydrate, i.e., if they lose water, they shrink and this is associated with the formation of echinocytes. This holds true albeit there are also other isovolumetric mechanisms to transform discocytes into echinocytes (Fischer, 2003). The well-known but mechanistically elusive “glass effect” belongs to this kind of transformation. The way around, if RBC overhydrate, the uptake of water swells the discocytes toward a sphere through intermediate forms of stomatocytes (Lim et al., 2002). From that point of view, the terminology of “dehydrated stomatocytosis” is indeed a contradiction in itself. The alternative denomination for “dehydrated stomatocytosis” is “xerocytosis,” indicating the occurrence of another pathophysiological cell shape, the xerocytes. This partly addresses the aforementioned contradiction but the alternative use of both terms, “dehydrated stomatocytosis” and “xerocytosis” adds to the confusion.

However, there is the concept of testing osmotic resistance with the ektacytometry as an incarnation that allows widely automated testing under reproducible and interlaboratory comparable conditions (Bianchi et al., 2015). This method became a kind of clinical standard and proved its usefulness in numerous hematological centers (Lazarova et al., 2017; Llaudet-Planas et al., 2018; Zaninoni et al., 2018; Vives-Corrons et al., 2021). In ektacytometry, a left shift of the osmotic fragility curve is compatible with a dehydration of the RBCs and due to differences in this curve, stomatocytosis was categorized into dehydrated and overhydrated stomatocytosis.

When comparing the two approaches, we favor the traditional cell shape-based concept because there is hardly any other option to explain the formation of stomatocytes, than overhydration. In contrast, a left shift of the curve in ektacytometry could have numerous explanations including the versatile composition of transmembrane transport proteins involved in volume regulation, such as the ion pumps Na^+^/K^+^-ATPase (Petrushanko et al., 2017), the Ca^2+^-ATPase (Dagher and Lew, 1988), ion channels such as TRPC6, Piezo1, the Gardos channel (Kaestner et al., 2020) or the recently described TRPV2 (Belkacemi et al., 2021), membrane transporters like band 3 protein (Bamberg and Passow, 1992) or the K^+^/Cl^−^-cotransporter (Adragna et al., 2006). Just to make it clear, we believe in the usefulness of ektacytometry as a diagnostic parameter for RBC-related diseases. It is only the strict terminological link to the hydration state that we like to discuss.

Although there is a deterministic relationship between hydration state and cell morphology in healthy RBCs as indicated by the SDE-scale (Lim et al., 2002), in the pathophysiological situations this relationship is widely distorted as the dehydration due to hemoglobinopathies indicate (Zaninoni et al., 2018; Krishnevskaya et al., 2021). Therefore, we believe the hydration state of the RBCs is an unsuitable parameter for disease classification.

## Discussion

The explanations above outline that a part of the nomenclature for rare anemias is not quite appropriate. The intention of this opinion paper is not to come up with a new terminology but to initiate the discussion about it. One possible and timely opportunity would be to orient the nomenclature of hereditary anemias on the mutation they are caused by, as it is done in other diseases, such as the “*VSP13A* disease,” which was proposed to replace “Chorea Acanthocytosis” (Walker and Danek, 2021). In hereditary dehydrated stomatocytosis/hereditary xerocytosis, we have partly experienced such a change. Mutations of the mechanosensitive ion channel Piezo1 were initially identified as the molecular cause for this disease (Zarychanski et al., 2012; Albuisson et al., 2013; Andolfo et al., 2013; Shmukler et al., 2014; Rotordam et al., 2018). Soon afterwards, a second molecular player was identified, namely the Gárdos channel (Andolfo et al., 2015; Glogowska et al., 2015; Rapetti-Mauss et al., 2015). However, it is increasingly recognized that the mutations of the Gárdos channel result in a distinct pathological phenotype compared to the mutations of Piezo1 and the former is now referred to as Gárdos channelopathy or *KCNN4* variants (Fermo et al., 2017, 2020). Ever since there is a kind of naming mess depending on the reporting authors, hereditary dehydrated stomatocytosis (hDSt) is differentiated into Piezo- or Gárdos-stomatocytosis, hDSt I and hDSt II or xerocytosis and Gárdos channelopathy, although this definition not always reflects the presence of stomatocytes or the dehydration state of the cell.

Also, in the case of hereditary spherocytosis we face two different problems: (i) sometimes spherocytes are not present and (ii) what we observe in blood films does not correspond to what we see in 3D (Figure 1). The 3D imaging approach helped us to realize that we need to think to a different nomenclature because morphology alone seems to be a limited diagnostic tool.

In any case, hereditary spherocytosis and hereditary stomatocytosis are presented here as examples to approach the problem. Hereditary spherocytosis because it is a well-known disease and among the rare anemias the most common one, with a clear diagnostic approach (Mohandas and Gallagher, 2008; Perrotta et al., 2008). However, in most centers of general medicine the first suspect of hereditary spherocytosis rises from the presence of spherocytes in the blood smear although it is known that the number of spherocytes may be low in most cases. On the contrary, the diagnosis of hereditary stomatosytosis was only based on morphological examination before the advent of the Next Generation Sequencing (NGS) technologies, and now we know that presence of stomatocytes may be associated to various different defects. Therefore, we are convinced the name “stomatocytosis” is an example that the nomenclature is, based on the current knowledge, an insufficient naming.

We admit that the naming is much less confusing when it comes to the enzymopathies, pyruvate kinase deficiency (Bianchi and Fermo, 2020) or G6PD deficiency (Luzzatto and Arese, 2018) name the defect protein.

We propose here to tackle the nomenclature of membranopathies by a holistic concept considering the entity of hereditary anemias and the genetic variants they are caused by. This initiative is meant to make a transition for the nomenclature of rare anemias. Although just for a few of them applies that “their names are just smoke and mirrors” the transition is meant to follow “Nomen est omen” for all of them.

## Author Contributions

All authors listed have made a substantial, direct and intellectual contribution to the work, and approved it for publication.

## Conflict of Interest

The authors declare that the research was conducted in the absence of any commercial or financial relationships that could be construed as a potential conflict of interest.
